# Age- and Sex-Dependent Effects of Moderate Exercise on Endogenous Pain Inhibition in Rats

**DOI:** 10.3390/biomedicines12051122

**Published:** 2024-05-18

**Authors:** Renan F. do Espírito-Santo, Sarah M. Margerison, Youping Zhang, Joshua Pak, Jin Y. Ro, Joyce T. Da Silva

**Affiliations:** 1 Department of Neural and Pain Sciences, University of Maryland School of Dentistry, Baltimore, MD 21201, USA; rfernandes@umaryland.edu (R.F.d.E.-S.); smargerison@som.umaryland.edu (S.M.M.); youzhang@umaryland.edu (Y.Z.); jpak@umaryland.edu (J.P.); jro@umaryland.edu (J.Y.R.); 2 Department of Physical Therapy and Rehabilitation Science, University of Maryland School of Medicine, Baltimore, MD 21201, USA

**Keywords:** pain, exercise, sex, age, testosterone

## Abstract

Diffuse noxious inhibitory controls (DNICs), or the pain inhibits pain phenomenon, refer to reduced pain-like behaviors that are displayed following a noxious conditioning stimulus located far from the test stimulus and have also been referred to as “descending control of nociception” when measured in awake-behaving animals. In this study, we sought to determine the impact of moderate long-term exercise on the DCN response and determine if this effect differed across age and sex. After a six-week exercise program consisting of 30 min of moderate treadmill running 5 days a week, the animals’ forepaws were injected with capsaicin, and DCN responses were assessed using thermal withdrawal latencies of the hind paw. Young, exercised male and female rats displayed prolonged DCN responses relative to their sedentary counterparts, with the young exercised male group displaying longer-lasting DCN facilitation than the young exercised females. Exercise did not impact DCN responses in either male or female aged rats. Additionally, the serum testosterone levels did not change following exercise in any group. Importantly, the levels of corticosterone did not change following the exercise program, indicating that changes in the DCN response are not due to stress-induced analgesia. Our findings suggest that moderate exercise can facilitate the DCN response in young animals, even when this exercise does not change the levels of serum testosterone.

## 1. Introduction

Diffuse noxious inhibitory controls (DNICs), or the pain inhibits pain phenomenon, are triggered by a noxious conditioning stimulus and result in reduced pain-like behaviors in response to a second noxious stimulus applied to other areas of the body. This effect is measured within the spinal cord at the level of individual neurons in anesthetized animals, where there is inhibition of wide-dynamic-range neurons in the dorsal horn by the application of a noxious stimulus outside the receptive field of the recorded neuron [1]. Recently, the term “descending control of nociception” (DCN) was introduced as a quantification of descending modulatory pathway activation upon conditioning in conscious animals [2], a concept that we have chosen to incorporate. The pathway that is responsible for DCN includes the rostral anterior cingulate cortex, the prelimbic cortex, and the insula at the cortical level; the thalamus sub-cortically; and the mesencephalic nucleus and periaqueductal gray in the midbrain [3]. It is mediated by mu-opioid receptor signaling in the anterior cingulate cortex [4] and serotonergic and noradrenergic systems in the spinal cord [5]. In humans, DNIC/DCN is often referred to as conditioned pain modulation (CPM) [6].

As in many measures of pain sensitivity, sex and age effects are apparent in the CPM or DCN response. In humans, many studies have shown a greater CPM effect in men relative to women [7,8]. Although preclinical studies using animal models do not precisely mimic human conditions and their translational potential is acknowledged, the same appears to be true in rats; male rats displayed a greater DCN response than both female rats and male gonadectomized rats [3]. While there are sex-specific mechanisms involved the development of chronic pain and persistent hyperalgesia, such as the neuroimmune mechanisms shown by microglial involvement in neuropathic pain models in males versus T-cell involvement in females, and the role of gonadal hormones [9], sex-based differences in analgesia that are secondary to exercise are less understood. Most preclinical research investigating the effects of exercise on pain-like behavior has used male rodents, and when both sexes have been used, results have been mixed, with some studies showing sex differences and others not [10]. DCN is also abolished with age; in a study comparing old and young rats, only young rats displayed a DCN response [11]. Data on CPM in humans appear to agree, showing a reduction in CPM in older adults [12,13].

Forced swimming, voluntary wheel running, and forced treadmill running have been used to measure the effects of exercise on pain in animal models, and all have advantages and drawbacks [10]. Forced swimming can increase the levels of cortisol [14]. This can be problematic, as stress itself can induce analgesia, and confound the analgesic effects of exercise [15]. Voluntary wheel running causes less stress; however, this paradigm requires specific equipment to record the number of wheel revolutions, and the amount of exercise performed by each animal cannot be precisely controlled, making the effect of exercise hard to measure [16]. Forced treadmill running avoids the problem of variable activity; however, this model has also been shown to increase biomarkers of stress [14]. Thus, it is important to measure these biomarkers in any forced exercise pain experiment.

The DCN response is increased after intense exercise in animals [17]. One recent study found that voluntary wheel running partially reduced dysfunction of DCN following an experimentally induced brain injury in rats [18]. However, little is known about the impact of moderate exercise on DCN and how this effect may vary by sex and age. Investigations of the effects of moderate exercise are important, as a moderate intensity paradigm is more clinically relevant for older adults; many older adults are not able to exercise at an intense pace. An exercise paradigm of 30 min of moderate exercise five days a week is consistent with the activity recommendations from the American Heart Association, the United States Department of Health and Human Services, and the World Health Organization [19,20]. Thus, it is important to understand the effects that this level of activity may have on endogenous pain-inhibitory mechanisms.

The goal of this study was to determine if a moderate level of exercise, as outlined above, alters the DCN response, and if this response differs across sex and age. We hypothesized that exercise will enhance DCN across all age and sex groups. We anticipated that the extent of this change would correlate with the serum testosterone levels. We expected the magnitude of the change in DCN after exercise to be associated with levels of serum testosterone.

## 2. Methods

### 2.1. Animals

Young (3–6 months old) and old (20–24 months old) male and female Fischer 344 rats were procured from the National Institute of Aging. Rats were housed in a temperature-controlled room with a 12 h light–dark cycle with ad libitum access to food and water. Throughout the study, the rats were continuously monitored to minimize unnecessary stress and discomfort, especially involving any aging-related health issues. The sample size ranged from 7 to 13 rats per group due to mortality in the older groups. All procedures were conducted according to the guidelines outlined in the National Institutes of Health Guide for the Care and Use of Laboratory Rats, as well as the standards set by the International Association for the Study of Pain. Additionally, these procedures were conducted in accordance with the Institutional Animal Care and Use Committee protocols approved by the University of Maryland. The rats were allowed a minimum of 7 days of acclimation in the housing facility before commencing the study.

### 2.2. Treadmill Exercise Protocol

**Figure 1:**
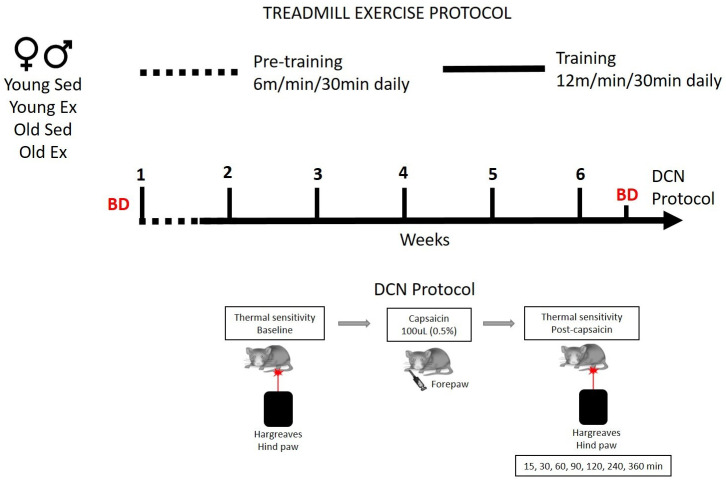
Treadmill exercise and DCN behavioral protocols. BD: blood draw, Ex: exercise, Sed: sedentary.

Age-matched male and female rats were randomly allocated to either the exercise or sedentary group. The groups were composed of the following categories: young males that exercised (YME), sedentary young males (YMS), young females that exercised (YFE), sedentary young females (YFS), old males that exercised (OME), sedentary old males (OMS), old females that exercised (OFE), and sedentary old females (OFS). The exercise group used a motorized treadmill for 30 min daily (Ugo Basile motorized treadmill, Gemonio, Italy). The treadmill exercise regimen consisted of a running speed of 6 m/min during the first week, followed by a running speed of 12 m/min for an additional five weeks (as illustrated in Figure 1). Each week consisted of Monday through Friday, excluding the weekend. Each running lane was equipped with a shock grid made of 3 mm bars spaced 8 mm apart at the bottom of the ramp. If a rat failed to maintain the assigned speed, it would eventually fall onto the shock grid, and receive a 333 ms (3 Hz) pulse of a single electric shock with a current of 0.5 mA. The treadmill was set at an inclination of 0°. Rats in the sedentary group were placed inside the treadmill lanes for an equivalent duration of time (30 min daily) over the same total period (6 weeks), but the treadmill machine remained turned off.

### 2.3. Behavioral Test

The assessment of DCN in rats has been detailed in our previous studies [3]. In brief, we measured hind paw withdrawal latencies in response to test stimulus of noxious thermal stimulation both before and after administering capsaicin, the conditioning stimulus, into the left forepaw two days after completing the exercise regimen (Figure 1). Prior to conducting the DCN assay, the rats were allowed to acclimate to the experimental room for 30 min daily over three consecutive days. Following this acclimation period, the rats were placed on an elevated glass surface and given 10 to 20 min to acclimate. To measure hind paw withdrawal latencies, we directed a radiant heat source to the plantar surface of the hind paw from underneath the glass floor. A motion detector stopped both the lamp and timer when the paw was withdrawn. The voltage of the bulb was adjusted to achieve an average paw withdrawal latency of 10 to 12 s in naive rats. We implemented a 20 s cutoff time to prevent tissue damage. For each hind paw, three trials were conducted with at least a 5 min interval between trials. The average of these trials was used as the final thermal paw withdrawal latency. We measured hind paw withdrawal latencies to the noxious heat test stimulus before and at 15, 30, 45, 60, 90, 120, 240, and 360 min after the administration of capsaicin. The increase in hind paw withdrawal latency following capsaicin treatment in the forepaw served as a measure of DCN. Capsaicin (obtained from Millipore Sigma, St. Louis, MO, USA) was dissolved in a solution comprising ethanol (20%), Tween 80 (7%), and PBS (70%). Capsaicin (0.5% in 100 μL) was administered intradermally into the left forepaw once using a 27-gauge needle.

### 2.4. Enzyme-Linked Immunosorbent Assay (ELISA)

Blood samples were obtained from the artery on the ventral aspect of the rat’s tail both at baseline and the next day upon completion of the exercise regimen. The rats were anesthetized with isoflurane (1.5–2%) for all blood collection procedures. These blood samples were collected between 12 pm and 3 pm, subsequently centrifuged to separate the serum, and then stored at −20 °C until the assay day. The concentrations of total free testosterone (ng/mL) and corticosterone (ng/mL) levels from the serum samples were evaluated using ELISA assay kits provided by Cayman Chemical Company, following the manufacturer’s instructions.

### 2.5. Statistical Analysis

Results were analyzed using the statistical analysis software GraphPad Prism 9. Two-Way Repeated Measures ANOVA with the Tukey or Sidak methods for correction of multiple comparisons was performed to determine significant treatment and time effects for each age and/or sex group, sedentary or exercised. Weight data underwent percentage transformation and normalization to baseline averages that were specific to each group, addressing baseline weight discrepancies that were attributable to sex and age variables. To assess the overall temporal changes in DCN responses, the area under the curve (AUC) was computed for each rat using the trapezoidal rule. Welch’s *t*-test was employed to compare AUC values across age and sex groups, stratified by exercise condition. Additionally, one-way ANOVA with the Tukey method for correction of multiple comparisons was applied to analyze baseline levels of corticosterone and testosterone across groups. Differences were considered statistically significant at *p* < 0.05, and the data were presented as mean ± standard error of the mean (S.E.M.).

## 3. Results

### 3.1. General Observations

**Figure 2:**
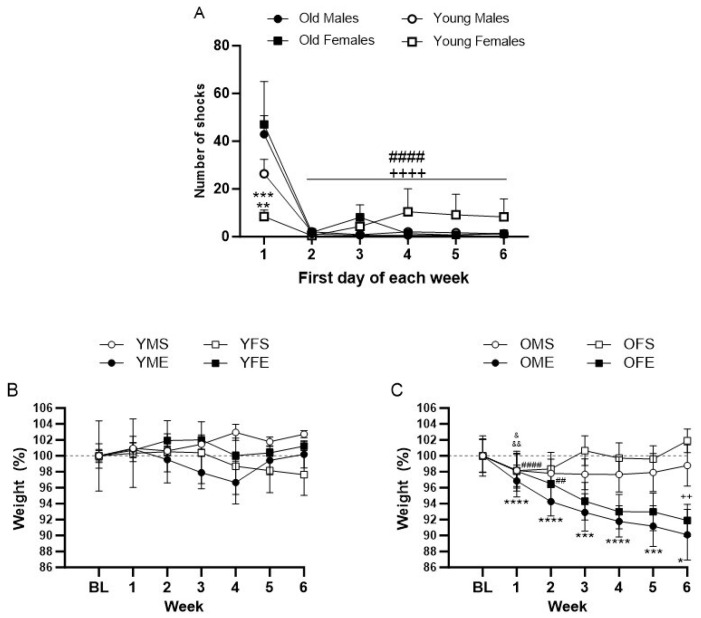
Number of shocks measured on the first day of each week of treadmill exercise protocol (**A**) and weekly weight variation in young (**B**) and old (**C**) sedentary and exercise groups. (**A**): ** *p* < 0.005 young females vs. old males, *** *p* < 0.0005 young females vs. old females; #### *p* < 0.0001 old males week 1 vs. weeks 2 to 6, and ++++ *p* < 0.0001 old females week 1 vs. weeks 2 to 6; no differences between week 1 and following weeks in young groups. (**B**): No differences between groups or time. (**C**): **** *p* < 0. 0001, *** *p* < 0. 0005, and * *p* < 0. 05 old males exercise vs. their baseline weight, #### *p* < 0.0001 and ## *p* < 0.005 old females exercise vs. their baseline weight, & *p* < 0.05 old males sedentary vs. their baseline weight, && *p* < 0.005 old females sedentary vs. their baseline weight, and ++ *p* < 0.005 old females exercise vs. old female sedentary at week 6. No differences between old males exercise and sedentary. Graphs represent mean ± SEM. Two-way RM ANOVA with Tukey’s correction. n = 7–13 rats per group. BL: baseline, YMS: young males sedentary, YME: young males exercise, YFS: young females sedentary, YFE: young females exercise, OMS: old males sedentary, OME: old males exercise, OFS: old females sedentary, OFE: old females exercise.

To gauge how animals across various age and sex groups adapted to the treadmill exercise protocol, we monitored the number of shocks administered to each animal throughout the experiment. These shocks were recorded during the exercise session on the first day of each week. During the first day of the exercise program (pre-training), all animals received shocks as they acclimated to the treadmill. Notably, older rats tended to receive more shocks than their younger counterparts, with older female rats (OF) experiencing a significantly higher number of shocks compared to their younger female counterparts (YF) (Figure 2A; *p* < 0.0005). Old male rats (OM) also received more shocks compared to YF at the first week (Figure 2A; *p* < 0.005). There was no statistical difference in the number of shocks between OM and YM, and YM and YF in the first week, and between any groups in the following weeks.

As the study progressed, the incidence of shocks decreased markedly in the old groups (Figure 2A; *p* < 0.0001). For both old groups (OM and OF), the number of shocks dwindled to nearly zero in the second week. The time comparisons considering week 1 versus each of the following weeks of exercise (weeks 2 to 6) showed that the number of shocks taken by the OM and OF groups reduced (Figure 2A; *p* < 0.0001). There was no statistical difference in the number of shocks between week 1 and the following weeks in the young groups.

The body weight of young animals who were exposed to daily treadmill exercise did not show a significant change over the course of the exercise (Figure 2B). In contrast, daily treadmill exercise significantly reduced the weight of old males and old females under exercise (OME and OFE). The weight loss was significant at the first week of exercise for OME and remained reduced at 6 weeks compared to their baseline weight (Figure 2C; **** *p* < 0. 0001, *** *p* < 0. 0005, and * *p* < 0. 05). The OFE rats exhibited weight loss compared to their baseline weight, but the difference was only statistically significant at weeks 1 and 2 (Figure 2C; #### *p* < 0.0001 and ## *p* < 0.005). There was a significant reduction in weight between OFE rats and their sex-matched sedentary group at week 6 (Figure 2C; ++ *p* < 0.005). There was no statistical difference in the weight between OME rats and their sex-matched sedentary group (OMS) at any time point. The weight of old sedentary rats was significantly different at week 1 relative to baseline (Figure 2C; & *p* < 0.05 for old males sedentary and && *p* < 0.005 for old females sedentary).

### 3.2. Effects of Treadmill Exercise on Endogenous Pain Inhibition

**Figure 3:**
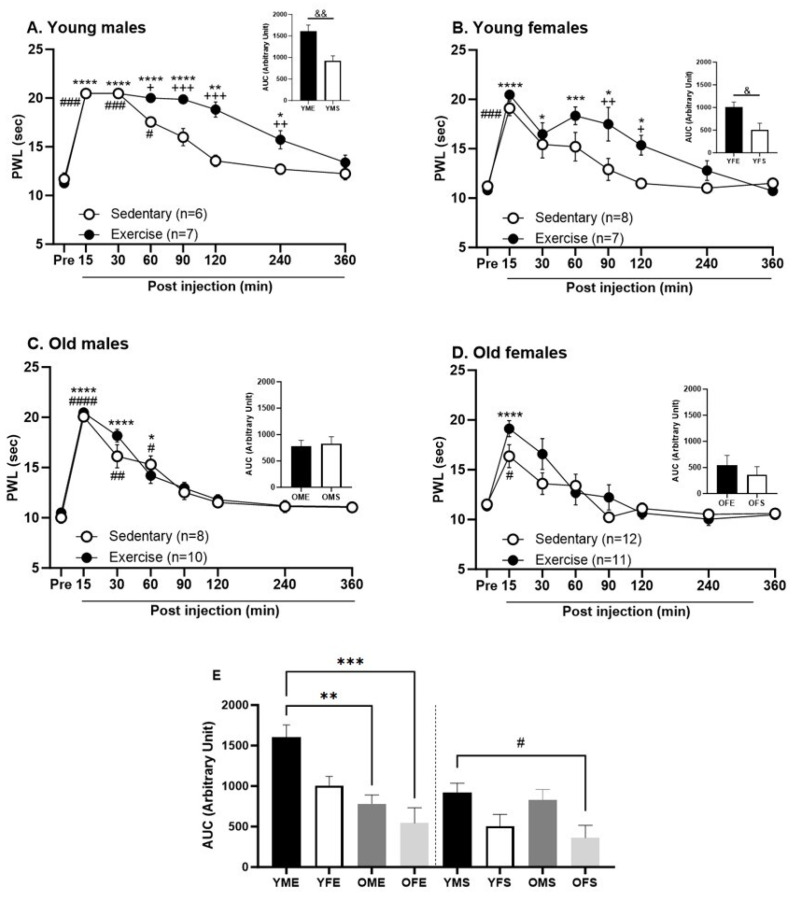
Exercise significantly enhances DCN responses in young males and females. Forepaw injection of capsaicin significantly increased mechanical thresholds of the hind paw of young males and young females that underwent treadmill exercise. DCN responses were significant compared to baseline in young males (**A**) and young females (**B**) with exercise **** *p* < 0.0001, *** *p* < 0.0005, ** *p* < 0.005, and * *p* < 0.05, and in sedentary young males (**A**) and sedentary young females (**B**) ### *p* < 0.0005 and # *p* < 0.05. Exercised young males (**A**) and females (**B**) exhibited higher DCN responses compared to sex-matched sedentary control groups (+++ *p* < 0.0001, ++ *p* < 0.005, and + *p* < 0.05 for group comparisons at different time points. && *p* < 0.005 and & *p* < 0.01 for group comparison using AUC). DCN responses were significant compared to baseline in old males (**C**) and old females (**D**) with exercise **** *p* < 0.0001 and * *p* < 0.05, and in sedentary old males (**C**) and old females (**D**) #### *p* < 0.0001, ## *p* < 0.005, and # *p* < 0.05. No group differences in DCN responses were observed in old males or old females with or without exercise (**C**,**D**). Bar graph shown in (**E**) compares AUC of overall magnitude of DCN responses within four exercised and four sedentary groups, respectively (*** *p* < 0.0005, ** *p* < 0.005, and # *p* < 0.05). Graphs represent mean ± SEM. Two-way RM ANOVA with Sidak’s correction was used for line graphs, and Welch’s *t* test or one-way ANOVA with Tukey’s correction were used for AUC graphs. Pre: pre-capsaicin injection, PWL: paw withdrawal latency, YMS: young males sedentary, YME: young males exercise, YFS: young females sedentary, YFE: young females exercise, OMS: old males sedentary, OME: old males exercise, OFS: old females sedentary, OFE: old females exercise.

To evaluate the impact of treadmill exercise on endogenous pain inhibition, we compared the DCN responses between exercised and sedentary rats across various sex and age groups. Previously, we reported that a capsaicin injection in the forepaw led to a substantial increase in hind paw withdrawal latencies to a noxious thermal stimulus, lasting approximately 60 min before returning to baseline within 120 min in healthy young male rats [11]. In this study, YMS rats exhibited a temporal DCN pattern that is qualitatively similar to that of naive young male rats [11]. However, it took 90 min for the PWL of YMS rats to return to baseline (Figure 3A; ### *p* < 0.0005 and # *p* < 0.05).

Young male rats subjected to treadmill exercise displayed prolonged DCN responses, with a significant increase in their paw withdrawal latencies relative to baseline, lasting up to 240 min post-capsaicin injection (Figure 3A; **** *p* < 0.0001, ** *p* < 0.005, and * *p* < 0.05). Group comparisons between YME and YMS show a significant effect of exercise on DCN responses from 60 to 240 min post-capsaicin injection, with the exercised rats showing a stronger effect (Figure 3A; +++ *p* < 0. 0001, ++ *p* < 0.005, and + *p* < 0. 05). The overall magnitude of DCN responses, measured as the area under the curve (AUC), was significantly greater for YME compared to YMS rats (Figure 3A; *p* < 0.005).

YFS rats demonstrated significant DCN responses for 15 min post-capsaicin injection only (Figure 3B; ### *p* < 0. 0005). Similar to the young males, exercise significantly improved their DCN responses for up to 120 min post-capsaicin injection (Figure 3B; **** *p* < 0.0001, *** *p* < 0.0005, and * *p* < 0.05). A significant group effect was observed at 90 and 120 min post-capsaicin injection (Figure 3B; ++ *p* < 0.005 and + *p* < 0. 05). Furthermore, there was a significant difference in the AUC between YFE and YFS rats, indicating a greater magnitude of DCN response in the exercised group (Figure 3B; *p* < 0.01).

Previously, we demonstrated that DCN responses are impaired in older animals [11]. In the current study, significant differences in paw withdrawal latency were observed across time in both the treadmill exercise and sedentary groups of old male and female rats (Figure 3C,D). OME and OMS rats demonstrated significant DCN responses from 15 to 60 min post-capsaicin injection (Figure 3C; **** *p* < 0.0001 and * *p* < 0. 05 for OME and #### *p* < 0.0001, ## *p* < 0.005, and # *p* < 0.05 for OMS), while OFE and OFS rats demonstrated significant DCN responses for only 15 min post-capsaicin injection compared to their pre-capsaicin measures (Figure 3D; **** *p* < 0.0001 for OFE and # *p* < 0. 05 for OFS). There was no significant difference in DCN responses between the treadmill exercise and sedentary groups in old male and female rats (group comparisons across time or AUC; Figure 3C,D).

The overall magnitude of DCN responses within the four exercised and four sedentary groups was compared using the AUC (Figure 3E). In the exercised groups, YME exhibited a higher magnitude of DCN response compared to the OME and OFE groups. There was no significant difference in the DCN response’s magnitude between YME and YFE, or between the other groups (Figure 3E). In the sedentary groups, YMS only exhibited a higher magnitude of their DCN response compared to the OFS group. There was no significant difference in the overall magnitude of the DCN response between the other sedentary groups (Figure 3E).

### 3.3. Sex and Age Differences in Corticosterone Levels Pre- and Post-Treadmill Exercise

**Figure 4:**
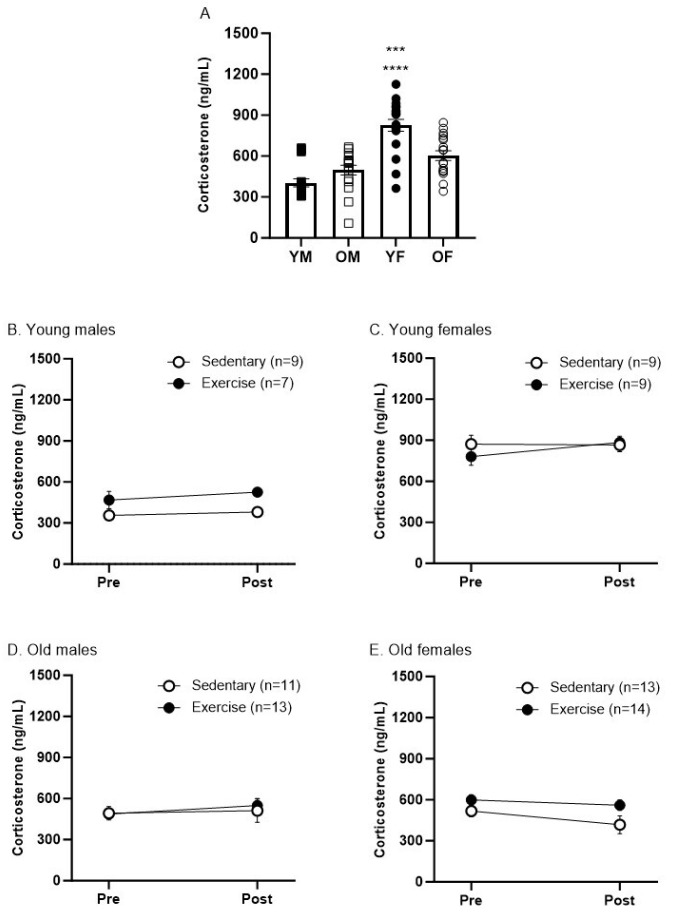
Baseline levels of corticosterone in young and old male and female rats (**A**). Graphs represent mean ± SEM. (**A**) One-way ANOVA with Tukey’s correction shows increased baseline corticosterone levels in young females vs. old females *** *p* < 0.05, and vs. old and young males **** *p* < 0.01. (**B**–**E**) There were no effects of the sedentary protocol or treadmill exercise on corticosterone levels using Two-way ANOVA with Sidak’s correction.

We evaluated the corticosterone levels pre- and post-treadmill exercise to investigate the potential effect of stress on rats undergoing exercise. First, we compared the baseline corticosterone levels of all groups by combining the data from both the sedentary and exercise groups for comparison. The baseline corticosterone level was significantly higher in young females compared to other groups, including old females and males in both age groups (Figure 4A; **** *p* < 0.0001 and *** *p* < 0.0005). There were no significant differences in corticosterone levels before and after treadmill exercise in any other groups (Figure 4B–E). Our findings suggest that the treadmill exercise protocol that we employed does not induce a significant level of stress.

### 3.4. Sex and Age Differences in Testosterone Levels Pre- and Post-Treadmill Exercise

**Figure 5:**
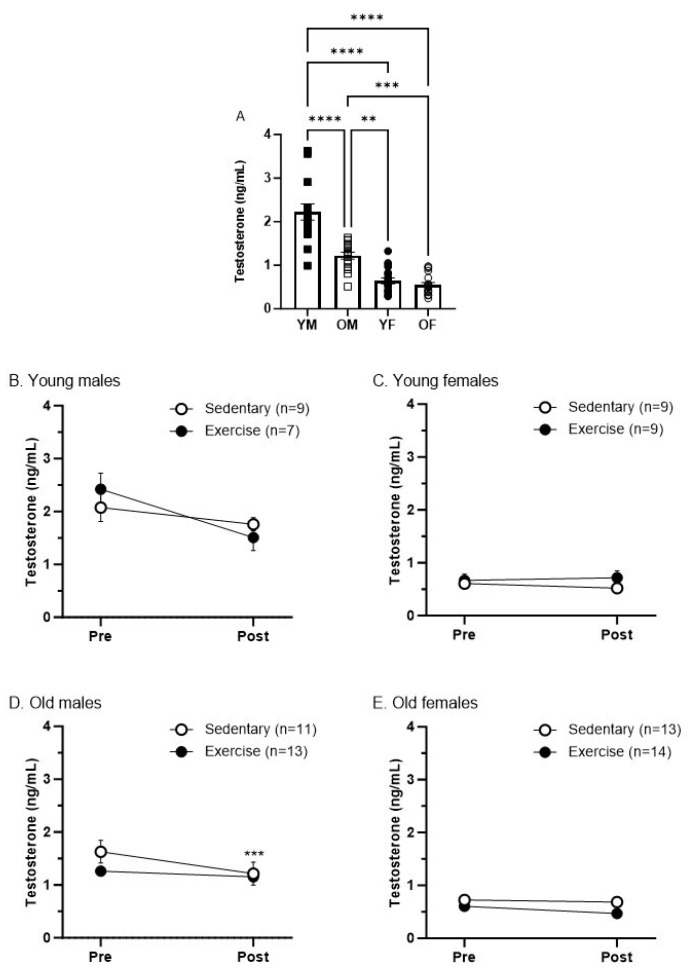
Baseline levels of testosterone in young and old male and female rats (**A**). Graphs represent mean ± SEM. (**A**) One-way ANOVA with Tukey’s correction shows increased baseline testosterone levels in young males vs. other groups **** *p* < 0.0001, and in old males vs. old females *** *p* < 0.0005 and vs. young females ** *p* < 0.005. (**B**,**C**,**E**) There were no effects of sedentary protocol or treadmill exercise on testosterone levels using Two-way ANOVA with Sidak’s correction. (**D**) There was a decrease in testosterone levels in sedentary old males at post-measure compared to pre-measure *** *p* < 0.0005.

We evaluated the plasma testosterone levels pre- and post-treadmill exercise to investigate whether our regimen of treadmill exercise alters testosterone levels and whether this varies by sex and age. First, as with the corticosterone, we compared the baseline testosterone levels of all groups by combining the data from both the sedentary and exercise groups. As expected, young male rats exhibited the highest levels of testosterone across all groups (Figure 5A; *p* < 0.0001). We observed higher testosterone levels in old male rats compared to young and old females (Figure 5A; *p* < 0.005 vs. young females, and *p* < 0.0005 vs. old females). There were no significant differences in testosterone levels between young and old female rats (Figure 5A).

There was a slight reduction in testosterone levels in OMS rats post-exercise compared to baseline (Figure 5D; *p* < 0.0005). There were no significant differences in testosterone levels within any groups before and after treadmill exercise or in young males, young females, and old females before and after the sedentary protocol (Figure 5B–E). Our findings suggest that the treadmill exercise protocol that we employed does not induce changes in testosterone levels.

## 4. Discussion

In this study, Fischer344 rats were exposed to a treadmill exercise protocol to determine if long-term, moderate exercise impacts DCN and if these effects are affected by sex and age. We found that DCN increased after moderate exercise in young but not old animals, which was contrary to our initially stated hypothesis. Additionally, this effect was more pronounced in the young male exercised group than in the young female exercised group. To control for effects of stress-induced analgesia on our findings, we also measured the levels of serum corticosterone at baseline and after completion of the exercise program. There were no differences in corticosterone levels within groups, indicating that we may ascribe the changes seen in DCN to exercise and not stress-induced analgesia. We also assessed the serum-free testosterone levels at baseline and after exercise to determine if the testosterone level impacted changes in DCN induced by exercise. However, contrary to our hypothesis, we found no changes in testosterone after exercise, except in the sedentary old male group, indicating that the effects of exercise on DCN that we observed are not related to serum-free testosterone levels.

Our primary finding is that our moderate exercise model increases DCN in young animals by increasing the duration of the analgesic response following administration of a conditioning stimulus (Figure 3). While there is support for the idea that intense exercise facilitates DCN [21], we are the first to show that moderate exercise may also have a facilitatory effect. In addition, we have found sex and age effects on exercise-facilitated DCN, where males displayed a greater effect than females, and no effect was noted in old rats of both sexes. This finding is consistent with the broader literature, both regarding DCN in animals [3] and CPM in humans [22]. While our findings discuss DCN responses facilitated by exercise, the current study was not a measure of exercise-induced analgesia (EIH). While EIH and DCN share similar mechanisms, including the release of serotonin [23,24] and endogenous opioids [25,26,27], as well as involvement of the rostral ventral medulla and periaqueductal gray [23,28], these processes are distinct. EIH is measured directly after exercise and does not require a conditioning stimulus (beyond the exercise itself) [29,30], while DCN is measured while the conditioning stimulus is applied. Additionally, EIH is short-lasting, resolving between 5 and 30 min after exercise cessation [29]. In contrast, the DCN effects observed in this study lasted as long as 240 min after the conditioning capsaicin injection in the young male group (Figure 3A).

DCN may be reduced with age, which is thought to be a result of a reduced efficacy of descending inhibitory systems [11,22,31,32,33]. It is worth noting that we have previously shown that DCN responses are impaired in old male and female rats compared to young groups [11]. In the current study, both the old male groups (exercised and sedentary) exhibited an overall magnitude of DCN responses like that of young males from the sedentary protocol (Figure 3E). Likewise, old females (exercised and sedentary) exhibited an overall magnitude of DCN responses that was comparable to that of young females from the sedentary protocol. Rats from the sedentary groups were placed inside the treadmill lanes for an equivalent duration of time (30 min daily) over the same total period (6 weeks). Thus, it is possible that the additional handling and transportation could affect DCN responses compared to our previous data, where rats had not undergone any treadmill protocol or sedentary control condition [11]. Despite a facilitation of DCN in young animals of both sexes under the exercise regimen, we did not see any change in the DCN response in old exercised animals. However, there are other possible explanations for the age-related differences seen in exercise-facilitated DCN following our protocol. In humans, muscle mass decreases with age in both sexes [34], and the composition of the muscle also changes, with an increased ratio of type 1 to type 2 muscle fibers, fewer total muscle fibers, and increased fatty infiltrate in the muscles of older adults [35]. This results in reduced force generation and increased difficulty with high-intensity exercise. Thus, it may be possible that our exercise protocol was more strenuous for old animals than for young. This idea is supported by the weight loss seen in the exercised older animals only, despite all animals having ad libitum food access. It is possible that this weight loss is related to a loss in muscle mass in the older animals due to the exercise program. In humans, older adults who lose weight quickly often lose lean body mass and muscle mass in addition to fat, especially when exercise only contains aerobic activity and no resistive training [36,37].

There is growing evidence indicating that CPM undergoes changes in the elderly population [13]. Given the close connection between altered CPM and various chronic pain conditions, it is reasonable to infer that impaired CPM may predispose older adults to an elevated risk of chronic pain issues. Numerous human studies consistently show that exercise has a moderate positive effect on chronic pain, irrespective of the exercise type or intensity level [38]. Research indicates that even a brief session of moderate-intensity exercise can enhance CPM in individuals with fibromyalgia, accompanied by heightened activity in brain regions involved in pain modulation [39]. This suggests that exercise might induce pain relief by engaging CPM circuits. However, it remains uncertain whether moderate exercise can effectively ameliorate chronic pain conditions by enhancing CPM. Our findings demonstrate that the exercise regimen that was effective for enhancing DCN in young animals did not produce the same results in older animals. These observations imply that exercise protocols tailored for younger populations for pain management may not be suitable for older individuals and highlight the need for the development of exercise programs specifically for older animals in future studies.

As sex effects have been noted in DCN [3,11], we suspected that testosterone levels may change following exercise and may mediate exercise-facilitated DCN. However, we saw no difference in testosterone levels from baseline to after completion of the exercise paradigm (Figure 5B–E). Other studies have shown differing findings relating to testosterone and exercise. In male rats with a deficiency in testosterone generation brought on by restricting food, an exercise program of 30 min of 15 m per minute treadmill running twice daily rescued testosterone generation [40]. However, in healthy male rats, vigorous exercise of 35 m per minute treadmill running at an eight percent incline for 1 h a day significantly reduced plasma testosterone for at least 6 h but less than 24 h [41]. It is possible that the lack of change in testosterone levels seen in this study was due to the moderate nature of our exercise program, as both mentioned studies included significantly more exercise in their models. However, despite seeing no change in testosterone levels following exercise activity, there was still a sex effect in the young male group, which was the group that displayed the highest levels of baseline testosterone (Figure 5A). It is possible that adequate baseline testosterone levels are required to maintain efficient DCN and to allow for exercise-facilitated DCN. The protective nature of testosterone against muscle pain has been shown in previous work [42].

This study had some limitations which reduce the weight of the inferences drawn from these findings. Our chosen exercise model was a moderately paced (12 m per minute) forced treadmill running program. To facilitate adherence to the program, these paradigms require an imperative stimulus, usually in the form of a noxious electric shock. While these programs allow for precise control of activity, they also may cause an increase in stress, creating a potential confounding factor in pain studies because stress may induce analgesia [14,15,43,44]. To address this potential concern, we measured the number of shocks received by each group on the first day of every week of training (Figure 2). While there was a difference in the number of shocks received during the first week of training, with the old male and female rats receiving significantly more shocks than the young female group, there were no differences in all the following weeks, indicating that between-group differences should not be confounded by the differing amounts of noxious stimuli received. We confirmed this by assessing the levels of serum corticosterone at baseline and upon completion of the exercise program (Figure 4). There was no effect of time on the serum corticosterone levels in any group, confirming that stress-induced analgesia as a mechanism for the facilitated DCN response following exercise in young animals is unlikely. Additionally, the confounding factor of weight on the older animal groups was also a significant limitation of this study; weight loss may play a role in the facilitation of DCN via exercise and may have impacted our results.

## 5. Conclusions

In summary, our study is the first to provide insights into the effects of moderate exercise on endogenous pain-inhibitory responses (DCN) and testosterone levels, as well as the role of sex and age in these outcomes. Our results indicate that moderate exercise can enhance the DCN response in young animals, even in the absence of changes in serum testosterone levels. The investigation of the effects of moderate exercise is especially important, as our paradigm closely resembles the exercise recommendations given by the World Health Organization and the Centers for Disease Control and Prevention [19,20]. Therefore, our results have direct relevance to human subjects. Future studies aimed at developing exercise programs tailored specifically for older animals, considering sex as a biological variable, are needed. Additionally, assessing the potential impacts of different exercise programs on testosterone levels, DCN responses, and pain-like behaviors remains a critical area of investigation.

## Data Availability

The data presented in this study are available on request from the corresponding author.
